# Efficacy and Safety of Emricasan in Liver Cirrhosis and/or Fibrosis

**DOI:** 10.6061/clinics/2021/e2409

**Published:** 2021-06-07

**Authors:** Li-ya Mu, Shu-qin Li, Li-xin Tang, Rui Li

**Affiliations:** Department of Gastroenterology, Heilongjiang Provincial Hospital, Harbin, Heilongjiang 150030, China

**Keywords:** Emricasan, Liver Cirrhosis, Liver Fibrosis, Caspase, Hepatic Function

## Abstract

This study aimed to perform a meta-analysis to determine the efficacy and safety of emricasan.

Nine databases were searched for clinical trials investigating the efficacy of emricasan treatment in patients with liver cirrhosis or fibrosis. A manual search was conducted to identify the missing trials. The quality of the included studies was assessed using the revised Cochrane risk of bias tool. Efficacy of emricasan treatment was defined as a positive change in apoptosis-related parameters from baseline to the last follow-up visit.

Overall, emricasan treatment is more effective in patients with liver cirrhosis or fibrosis than placebo (standardized mean difference [SMD] [95% confidence intervals (CI)]=0.28 [0.14; 0.41]). No significant change in model for end-stage liver disease (MELD) score between the emricasan and placebo groups was noted (SMD [95% CI]=0.18 [-0.01; 0.36]; *p*=0.058). A 50 mg dose of emricasan had the highest efficacy rate compared to placebo (SMD [95% CI]=0.28 [0.06; 0.50]; *p*=0.012), followed by the 5 mg dosing regimen (SMD [95% CI]=0.28 [0.06; 0.50]; *p*=0.012). Treatment with emricasan resulted in significant reductions in ALT (mean difference (MD) [95% CI]=-5.89 [-10.59; -1.20]; *p*=0.014) and caspase3/7 levels (MD [95%CI]=-1215.93 [-1238.53; -1193.33]; *p*<0.001), respectively. No significant increase in the rate of overall adverse events was noted (OR [95% CI]=1.52 [0.97; 2.37]; *p*=0.069).

Treatment with emricasan is more effective in improving liver function and apoptosis parameters compared to placebo, with a well-tolerated safety profile. However, due to the poor quality of the analyzed studies, the small number of trials and patients, and the short follow-up periods, more robust trials are still warranted.

## INTRODUCTION

Chronic liver diseases pose a major global health problem, accounting for approximately 2 million deaths per year worldwide (1). Many underlying etiologies have been identified, including viral hepatitis (hepatitis B virus [HBV] and hepatitis C virus [HCV]), alcoholic steatohepatitis, non-alcoholic steatohepatitis (NASH), autoimmune disorders, and genetic diseases. Organ fibrosis is a hallmark of disease progression in chronic inflammatory diseases and contributes to 45% of all-cause mortality globally (2). Similarly, the development of hepatic fibrosis is a significant determinant of quality of life and prognosis (3). Therefore, the degree of liver fibrosis correlates with liver function and is a major risk factor for hepatocellular carcinoma (4). Chronic portal hypertension secondary to hepatic fibrosis is the main cause of clinical complications, such as hydropic decompensation, bleeding events, and hepatic encephalopathy (3). As a result, hepatic cirrhosis is currently recognized as the eleventh most common cause of mortality worldwide (1) and the fourth most frequent cause of mortality in central Europe (5,6).

Hepatic fibrosis is mainly characterized by the buildup of the extracellular matrix, where its accumulation leads to the destruction of the physiological architecture of the liver (7). Various toxic, metabolic, and viral diseases act mainly by damaging hepatocytes, with subsequent infiltration of immune cells. This leads to the activation of trans-differentiation of hepatic stellate cells (HSCs) into collagen-producing myofibroblasts (8,9). Since hepatocellular death is the major trigger of inflammation, HSC activation, and fibrosis of all etiologies (10,11), the inhibition of hepatocyte apoptosis would reduce the activation of HSCs in liver fibrosis (12-14).

Caspases, a family of eleven intracellular cysteine proteases, have recently been recognized for their role in mediating apoptosis and regulating inflammatory and immune responses in apoptotic cells (15,16). Caspases 3, 6, and 7 (executioner caspases) (16) cleave many cell proteins (*i.e.*, cytokeratin-18 (CK-18)) and mediate the production of proinflammatory, profibrotic hepatic microvesicles (17). These microvesicles interact with hepatic stellate/myofibroblasts, as well as endothelial cells living liver sinusoids (17), resulting in the activation, migration, and genetic expression of fibrosis (18). Meanwhile, inflammatory caspases, or caspases 1, 4, and 5 (16), act by activating interleukin-1 (IL-1) (15), while initiator caspases (*i.e.*, caspases 2, 8, 9, and 10) (16) play a critical role in priming the NLRP3 inflammasome and producing IL-1 β. Therefore, inhibition of caspases may be beneficial in the management of liver fibrosis and cirrhosis.

Emricasan, or IDN-6556, is an oral pan-caspase inhibitor. It has been reported to reduce liver apoptosis, inflammation, and fibrosis in animal models of liver injury, including NASH (19) and carbon tetrachloride (CCL_4_)-induced cirrhosis (20). Moreover, it has been reported that emricasan reduces excessive caspase activity, as well as alanine aminotransferase (ALT), in patients with hepatitis C (21,22) and NASH (23). Several randomized placebo-controlled clinical trials have been conducted to study the efficacy of emricasan treatment in patients with hepatic fibrosis (24) or cirrhosis (25). One study using emricasan revealed a reduction in hepatic venous pressure gradient (HVPG) in a group of patients with NASH cirrhosis (25). In contrast, another trial noted that emricasan did not have a beneficial impact on inflammation and fibrosis in patients with NASH-associated F1-F3 fibrosis (24). Therefore, we conducted this systematic review and meta-analysis to determine the efficacy and safety profile of emricasan (IDN-6556) in improving hepatic function, caspase-related biomarkers, and fibrosis/cirrhosis in patients with liver fibrosis or cirrhosis.

## MATERIALS AND METHODS

### Search strategy and study selection

The study process followed the accepted methodology recommendations of the PRISMA checklist for systematic review and meta-analysis, in which registration of the protocol was not required (26). A systematic electronic database search was conducted for relevant studies published from inception until May 2, 2020 in nine databases, including Google Scholar, System for Information on Grey Literature in Europe, Scopus, Web of Science (ISI), PubMed, Virtual Health Library, Clinical Trials.gov, metaRegister of Controlled Trials (mRCT), and the WHO International Clinical Trials Registry Platform databases using keywords, medical subject (MeSH) terms, and publication types based on the PICO framework (participants, comparison, intervention, and outcomes). The participants were patients with liver cirrhosis and/or fibrosis who were treated with IDN-6556/PF-03491390 (emricasan), while the comparison group was the placebo or control group, and all relevant efficacy and safety outcomes were included. Efficacy was defined as a change (increase or decrease) from baseline to the last follow-up visit. In the case of multiple outcomes, efficacy was measured by the change in the outcome, which was reported as the main outcome of the study or the most relevant outcome of the cirrhosis/fibrosis measures (to maintain homogeneity).

We further performed a manual search of references in our included papers to avoid missing relevant studies (27,28). We included all original studies that assessed the efficacy and safety of emricasan in patients with liver cirrhosis and/or fibrosis. Papers were excluded if at least one of the following exclusion criteria was identified: non-original studies; non-human (*in vitro* or animal) studies; duplicate records; overlapping data; studies with data that could not be reliably extracted or were incomplete; abstract-only articles; and reviews, theses, books, conference papers, or articles without available full texts (conferences, editorials, author response, letters, and comments). The title and abstract screenings were performed by four independent reviewers. Three independent reviewers performed full-text screening to ensure the inclusion of relevant papers in our systematic review. Any disagreement was resolved by discussion and referring to the senior author when necessary.

### Data extraction

Three authors developed a data extraction sheet using Microsoft Excel. Data extraction was performed by three independent reviewers using Microsoft Excel software. The fourth independent reviewer verified the data to ensure accuracy of the extracted data. All disagreements and discrepancies were resolved by discussion and consultation with the senior author when necessary.

### Quality assessment

Three independent reviewers evaluated the risk of bias in the included studies. The revised Cochrane quality assessment tool was used to determine the quality of the randomized studies (29). Any discrepancies between the reviewers were resolved through discussion.

### Statistical analysis

All data were analyzed using R software version 4.0.0 (30). For all outcomes, the “meta” package was used to analyze the change from baseline for both intervention and control groups and to compute the standardized mean difference (SMD) or mean difference (MD) and the corresponding standard errors (SE) (31). For easier interpretation, results were standardized to be in a positive direction, with details regarding reductions or increases provided in the results. The SMD was used to assess the main efficacy outcome due to the difference in measurement methodology among the included studies (32,33). The corresponding 95% confidence intervals (CIs) of the pooled effect sizes were calculated. To assess safety (the rates of different adverse events), odds ratios (ORs) and their corresponding 95% CIs were calculated.

For different follow-up visits, the last visit and/or the visit with the most complete data were used in the analysis. Heterogeneity was assessed using Q statistics and I^2^ test, and the analysis was performed using the fixed-effects model due to the absence of significant heterogeneity among the included studies (34,35). Publication bias could not be assessed using Egger’s regression test due to the small number of included studies (less than 10) (36,37).

## RESULTS

### Search results

**Figure 1:**
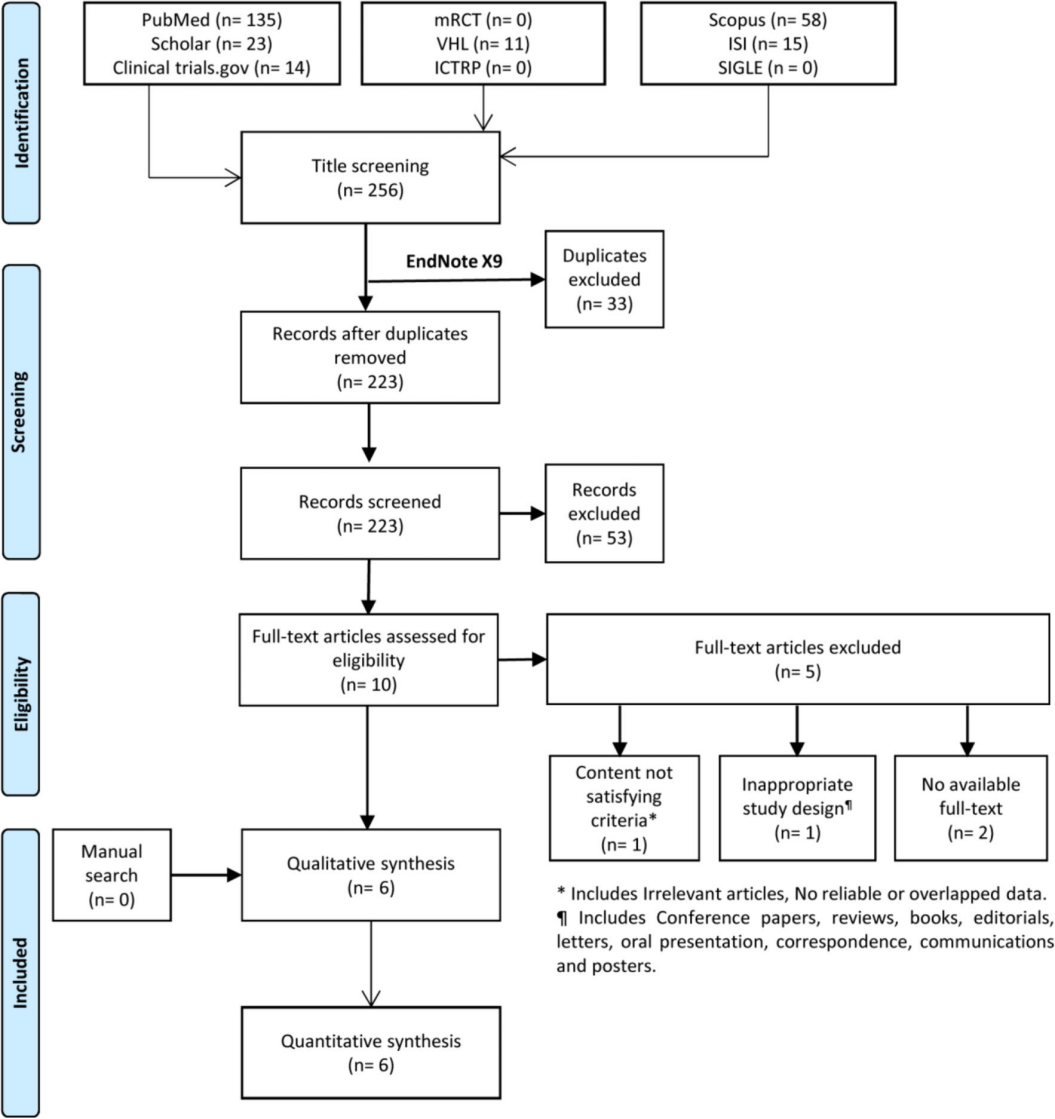
PRISMA Flow diagram of the search and screening process.

We identified 256 records after excluding 33 duplicates using Endnote X9 software. Title and abstract screening resulted in ten records eligible for further full-text screening. No papers were added after performing the manual search trials. In the end, we included six studies in our systematic review and meta-analysis (Figure 1).

### Study characteristics and quality of the included studies

All included papers were randomized trials, and all studies were placebo-controlled, except for one single-arm study (25). The sample size of the included studies ranged from 23 (25) to 318 patients (24). Also, follow-up durations were variable and ranged from 28 days (25) to 76 weeks (24). In addition, emricasan doses were variable and ranged from only 5 mg twice daily (21,24,38) to up to 400 mg thrice daily (21) (Table 1).

**Figure 2:**
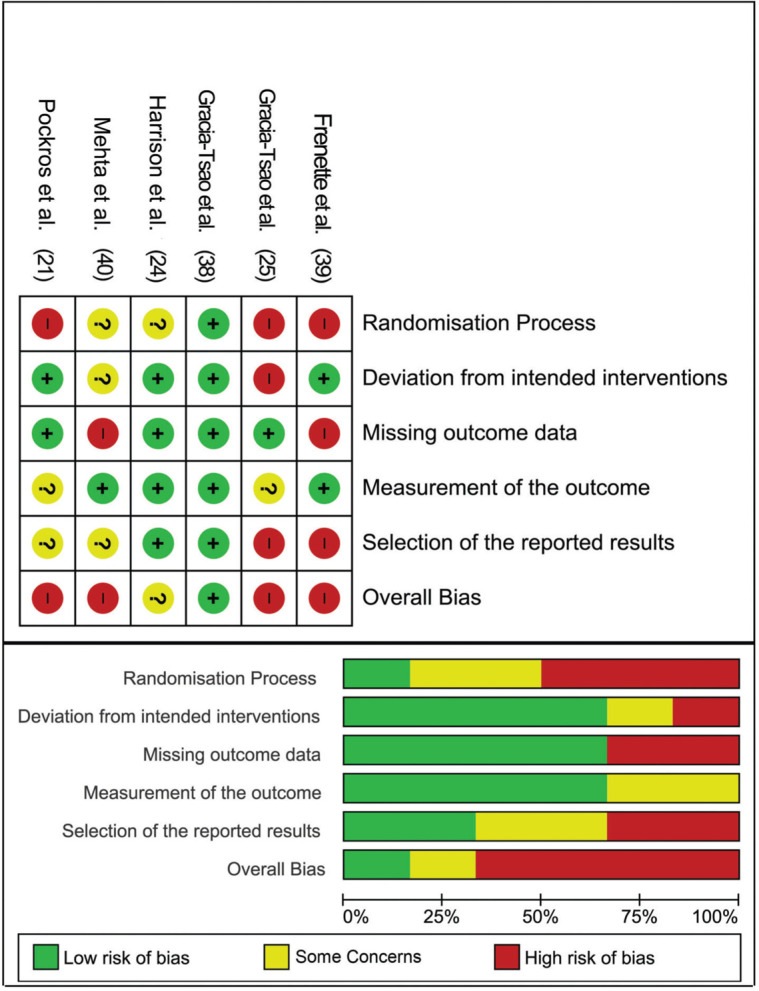
Quality assessment of the included studies.

In terms of quality assessment, four papers (21,25,39,40) had an overall high risk of bias, while one raised some concerns (24), and the last study showed a low risk of bias (38). The high risk of bias was mainly detected in the randomization process, missing outcome data, and selection of the reported results (Figure 2).

### Assessment of efficacy

**Figure 3:**
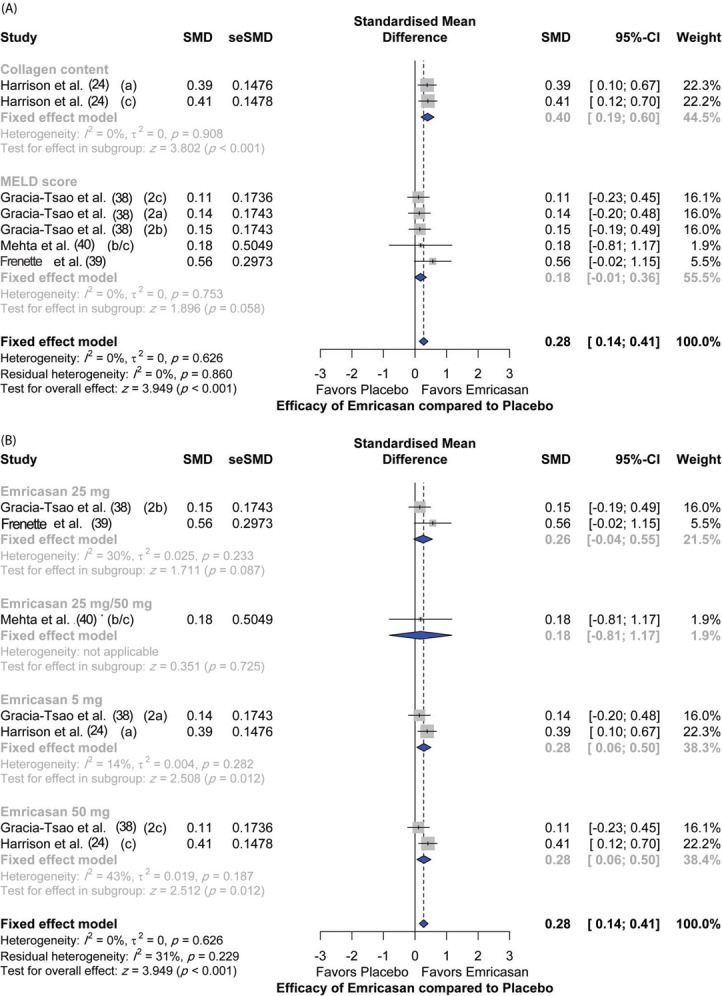
Main measures of Emricasan efficacy compared to placebo. (A) Assessed outcomes; (B) Dosing regimens: a=5 mg; b=25 mg and c=50 mg.

Four studies assessed the efficacy of emricasan in comparison to placebo, with a total of 692 patients. There was an overall significant (*p*<0.001) efficacy reported in the treatment group compared to the placebo group (SMD [95% CI]=0.28 [0.14; 0.41]). In terms of single outcomes, emricasan treatment showed significant efficacy in increasing liver collagen at different doses (SMD [95% CI]=0.40 [0.19; 0.60]; *p*<0.001); however, there was no significant effect on the model for end-stage liver disease (MELD) score (SMD [95% CI]=0.18 [-0.01; 0.36]; *p*=0.058). Moreover, there was no significant heterogeneity among the included studies (I^2^=0%; *p*=0.626) (Figure 3A).

In terms of dose regimens of emricasan, 50 mg doses showed the highest efficacy compared to placebo (SMD [95% CI]=0.28 [0.06; 0.50]; *p*=0.012), followed by the 5 mg dosing regimen (SMD [95% CI]=0.28 [0.06; 0.50]; *p*=0.012). In contrast, emricasan 25 mg and emricasan combined 25/50 mg did not show significant efficacy compared to placebo [(SMD [95% CI]=0.26 [-0.04; 0.55]; *p*=0.087) and (SMD [95% CI]=0.18 [-0.81; 1.17]; *p*=0.725), respectively] (Figure 3B).

**Figure 4:**
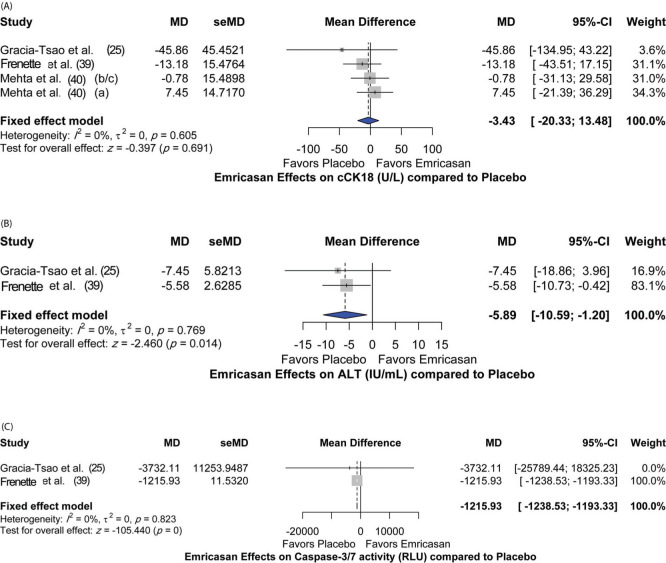
Other measures of Emricasan efficacy compared to placebo. (A) Cleaved cytokeratin 18; (B) Alanine aminotransferase (IU/mL); (C) Caspase 3/7 activity (RLU): a=5 mg; b=25 mg and c=50 mg.

Additionally, the effect of emricasan treatment on different parameters was assessed. Different doses of emricasan did not show a significant effect on reduction of cleaved cytokeratin 18 (cCK18) (MD [95%CI]=-3.43 [-20.33; 13.48]; *p*=0.691) compared to placebo (Figure 4A). Nevertheless, significant reductions in alanine aminotransferase (ALT) and caspase3/7 levels were detected [MD 95%CI=-5.89 [-10.59; -1.20]; *p*=0.014) and (MD [95%CI]=-1215.93 [-1238.53; -1193.33]; *p*<0.001), respectively] (Figure 4B and 4C). There was no heterogeneity among the included studies for all the assessed parameters, with I^2^=0% and *p*-value >0.05.

### Safety outcomes

**Figure 5:**
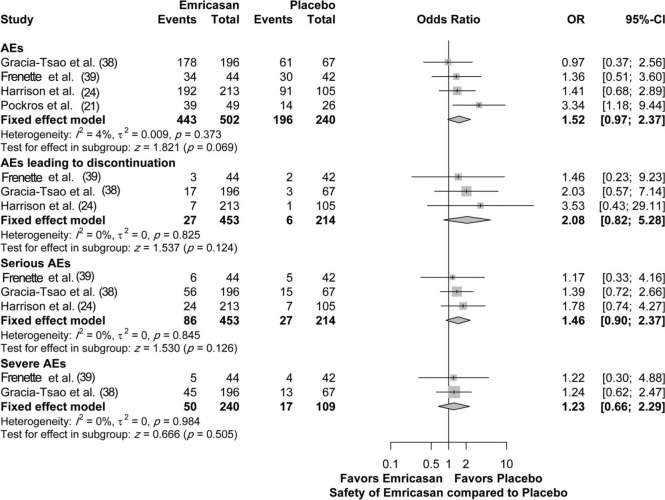
Safety of emricasan treatment (all doses) compared to placebo.

Four studies assessed the safety of emricasan compared to placebo, with a total of 742 patients. There was no significant increase in the adverse event (AE) rate, regardless of the overall AEs (OR [95% CI]=1.52 [0.97; 2.37]; p=0.069), serious AEs (OR [95% CI]=1.46 [0.90; 2.37]; *p*=0.126), severe AEs (OR [95% CI]=1.23 [0.66; 2.29]; *p*=0.505), or AEs leading to discontinuation (OR [95%CI]=2.08 [0.82; 5.28]; *p*=0.124). There was no heterogeneity among the included studies for all the assessed parameters, with I^2^=0% (4% for overall AEs) and *p*-value >0.05 (Figure 5).

## DISCUSSION

Progressive chronic liver disorders, including non-alcoholic steatohepatitis (41), hepatitis C (42), hepatitis B (43), and alcoholic liver diseases (43), have all been reported to be correlated with excessive caspase activation and liver cell apoptosis. In this context, caspase inhibitors, particularly pan-caspase inhibitors, have been shown to have a protective effect against hepatocyte injury in animal models of liver failure secondary to alcoholic cirrhosis, fatty liver diseases, and cholestatic liver disease (44-46). In these models, caspase inhibitors have been shown to have a significant effect in attenuating inflammation and fibrosis. Subsequently, several single-arm and placebo-controlled clinical trials were conducted in patients with cirrhosis and fibrosis associated with different underlying etiologies. Therefore, we conducted this meta-analysis to gather data presented in each individual trial to determine the efficacy and safety of emricasan, a pan-caspase inhibitor, in improving liver injury (related to caspase activation), apoptosis markers, and clinically associated parameters.

In our systematic review, a total of four studies included patients with evident cirrhosis (radiologically, clinically, or biochemically) (25,), while two studies included patients with different degrees of fibrosis (F1-F3 based on CRN fibrosis staging) but without cirrhosis (21,24). Overall, we found that all dose regimens of emricasan were significantly more effective compared to placebo. This finding was based on analysis of four trials (692 patients), with no heterogeneity among the included studies. In terms of single outcomes, we found that different doses of emricasan were associated with a significant increase in liver collagen content compared to placebo. Moreover, we noted no significant change in the MELD score between emricasan at various doses and placebo among all included patients. Notably, the study by Frenette et al. (39) with 86 patients with cirrhosis of various etiologies and MELD scores ranging from 11 to 18, revealed that the subgroup of subjects with MELD scores ≥15 had significant improvement in MELD scores after 3-6 months of treatment with 25 mg emricasan. This variation in the response between the low- and high-MELD score groups could be explained by the fact that the total bilirubin levels and international normalized ratio (INR) values were much higher in patients with MELD scores ≥15 compared to those with low MELD scores (<15); therefore, a significant improvement in liver function would be easier to detect. Furthermore, Frenette et al. (39) included patients with various causes of cirrhosis (namely, alcoholism, NASH, and hepatitis C); therefore, the insignificant change in MELD scores among all included patients could be related to the wide diversity of causes of cirrhosis.

Since liver function and portal hypertension are the two critical components of end-stage liver disease, it is important to discuss changes in HVPG following emricasan treatment in patients with cirrhosis. This factor was studied in only two trials (a single-arm and a placebo-controlled study), and thus, this parameter could not be used in our meta-analysis. However, it should be noted that patients with cirrhosis with Child-Pugh class A score and severe portal hypertension (HVPG >12 mmHg) have shown significant, clinically meaningful reductions in HVPG within 28 days of emricasan treatment (25). Meanwhile, Garcia-Tsao et al. (38) conducted a trial with 318 patients with NASH CRN F1-F3 fibrosis stage who were treated with emricasan (5 or 50 mg twice daily). However, the authors noted that emricasan failed to significantly reduce mean HVPG compared to placebo after 48 weeks of treatment. It should be noted that while improvement in clinical parameters and liver function may be achieved shortly after treatment, regression of cirrhosis and improvement in portal hypertension (reduction in HVPG) may take years. Therefore, more trials with longer treatment duration and longer follow-up periods are warranted to determine the effect of emricasan in treating portal hypertension in patients with cirrhosis.

In our meta-analysis, we also noted that different doses of emricasan (5-50 mg) were associated with a significant reduction in alanine aminotransferase (ALT) and executioner caspases (caspases 3/7). The reduction in ALT was evident shortly after a brief period of treatment (14 days) with emricasan among 105 patients with NASH fibrosis (F0-F3) and elevated ALT levels at baseline (21). It is presumed that this rapid reduction in ALT levels could be related to the anti-apoptotic effect of emricasan on hepatocytes, and, thus, preventing the release of ALT into the circulation; however, the validity of this hypothesis requires further investigation. In the case of chronic liver disease, apoptosis may occur secondary to death receptor signaling, particularly through the activation of the Fas pathway (47-49). In this context, emricasan has been shown to be effective in blocking Fas-induced cell apoptosis *in vitro* and in animal studies (45,50). Therefore, reduction in ALT levels could be explained, in part, by reduction in Fas-induced hepatocellular death. Meanwhile, in the study by Harrison et al. (24), serum levels of ALT decreased markedly during the first 4 weeks of treatment, with the effect being more pronounced in the 50 mg dosing group than in the 5 mg dosing group. However, ALT levels tended to reach baseline at 72 weeks. The same was noted for caspases 3/7 (24). Caspases 3/7 normally cleave keratin-18 into cleaved cytokeratin-18 (cCK-18) during apoptosis, which can be detected by the M30 monoclonal antibody (51). Meanwhile, total cellular death can be measured by levels of full-length cytokeratin-18 (flCK18)/M65, which has the ability to detect both cCK-18 and intact keratin-18 (52). However, upon analyzing emricasan at different doses, we did not find a significant change in cCK-18 levels compared to placebo. However, in the study by Frenette et al. (39), it was noted that cCK-18 and flCK-18 were significantly lower than placebo in the subgroup of patients with alcoholic cirrhosis, while no significant change was observed in the other subgroups (NASH and hepatitis C-related cirrhosis). Therefore, it is possible that the various etiologies of cirrhosis in the analyzed studies could have obscured the treatment effects.

Various doses of emricasan have been studied in clinical trials, ranging from 5 mg to 400 mg twice or thrice daily. In terms of dosing, emricasan 50 mg showed the highest efficacy compared to placebo, followed by emricasan 5 mg. However, emricasan doses of 25/50 and 25 mg did not show significant superiority over placebo in terms of efficacy. In the trial with the highest sample size (318 patients), it was noted that both doses of 5 and 50 mg had evident biological effects; however, the 50 mg dose had significantly more pronounced effects related to reduction of serum ALT, executioner caspases, and cCK-18 (24). Moreover, emricasan 50 mg was the only dosing regimen that markedly reduced M30 levels, an apoptosis marker (40). However, the 50 mg dose did not significantly improve the MELD score, CLIF-C ACLF score, or CLIF-C organ function. This could be related to the short duration of drug administration (14 days) and the small sample size (low power) in that trial (23 cases).

Overall, treatment with emricasan at various doses was generally well-tolerated in most studies. Overall, in our meta-analysis, we noted no significant increase in emricasan-related AEs compared to placebo. Moreover, no significant increase in serious AEs, severe AEs, and AEs that led to discontinuation were observed compared to placebo. The observed complications were those typically noted in patients with decompensated cirrhosis (39). The most commonly reported AEs in patients treated with emricasan at all doses included the following: headache (15.9%), nausea (15.9%) (39), diarrhea (16%), upper respiratory tract infection (10.3%) (24), peripheral edema (15.8%) (38), and abdominal pain (8%) (21).

Although the results of our meta-analysis provide helpful insights into the therapeutic potential of emricasan in improving liver function, clinical parameters, and apoptosis markers in patients with hepatic cirrhosis or fibrosis with or without complications, our results should be interpreted with caution. The number of available trials in the literature is limited, and more trials with larger sample sizes and longer follow-up durations are warranted. Furthermore, the primary outcome endpoint of each individual trial was different, with many variables not included in the meta-analysis due to unavailability of relevant data in more than one trial. It is unclear at this time whether the same trends will be observed in a meta-analysis based on a greater number of studies. Although we noted no significant heterogeneity among the analyzed studies, the reported 95% CI were quite wide in almost all the analyzed parameters, indicating a significant degree of uncertainty regarding the findings. Moreover, more than half of the included trials had a high risk of bias, with one trial having some concerns regarding study design and patient randomization. Finally, more robust randomized controlled trials are needed to reach definitive conclusions regarding the efficacy of emricasan in improving apoptosis-related parameters among patients with liver cirrhosis or fibrosis.

## CONCLUSIONS

Emricasan is more effective compared to placebo in improving apoptosis-related parameters, executioner caspases, and clinical parameters, such as serum ALT levels. No significant improvement in the MELD or cCK-18 levels was noted. Emricasan 50 mg has superior efficacy over other dosing regimens (5, 25, and 25/50 mg). Treatment with emricasan is well-tolerated, with no significant increase in the rate of AEs compared to placebo. However, more robust, placebo-controlled clinical trials, with larger sample sizes and longer follow-up periods, are needed to verify the efficacy of emricasan in patients with liver fibrosis/cirrhosis.

## AUTHOR CONTRIBUTIONS

Mu LY wrote the manuscript. Li SQ and Tang LX collected and analyzed the data. Li R approved the manuscript. All of the authors have read and approved the final version of the manuscript.

## Figures and Tables

**Table 1:** Characteristics of included studies.

Citation	Sample size	Study design	Intervention arm	Control arm	Inclusion criteria	Exclusion criteria	Follow-up period	Losses to follow-up	Primary outcome	Secondary outcomes
**Frenette et al. (39)**	86	A randomized, placebo-controlled trial	Emricasan 25 mg/ twice daily for 3 months	Placebo twice daily/3 months	Patients were Child-Pugh class A or B with compensated or decompensated cirrhosis (clinical, radiological, or biochemical evidence), with MELD scores ranging from 11 to 18.	Autoimmune hepatitis, active inflammatory bowel disease, hepatitis B-infected subjects on treatment for <3 months, hepatitis C-infected subjects planning to receive anti-hepatitis C virus (HCV) treatment, Child-Pugh class C, international normalized ratio (INR)≥2.5, platelets ≤20 x 10^9^/L, hepatic encephalopathy grade III, serum creatinine ≥2 mg/dL, alcohol consumption >21 oz/week for males or 14 oz/week for females, variceal hemorrhage within 3 months of screening, and uncontrolled ascites	3 months (primary outcome assessment point)	12 cases (13.95%): Intervention (4 cases), and Placebo (8 cases)	Emricasan reduced cCK-18 by -13% relative to placebo at 3 months (*p*=0.092)	1- Emricasan significantly reduced serum levels of flCK-18 (P=0.02) and caspase (P<0.001) at 3 months compared to placebo. 2- No significant differences between Emricasan and placebo in mean MELD (P=0.466) or Child-Pugh scores (P=0.124) at 3 months. 3- No differences between Emricasan and placebo regarding total bilirubin, INR, or serum albumin at 3 months.
**Garcia-Tsao et al. (38)**	263	A randomized, placebo-controlled trial	Emricasan (5, 25, and 50 mg) twice daily for 48 weeks	Placebo	Patients with cirrhosis due to non-alcoholic steatohepatitis (NASH) and baseline HVPG ≥12 mmHg.	Other causes of cirrhosis, compensated or decompensated (no more than one decompensating event)	48 weeks	13 cases at 24 weeks and 44 cases at 48 weeks	There were no significant differences in ?HVPG for any emricasan dose (5, 25, 50) *vs.* placebo (- 0.21, -0.45, -0.58 mmHg, respectively)	1- Biomarkers decreased significantly with emricasan at week 24 but returned to baseline levels by week 48. 2- New or worsening decompensating events (∼10% over median exposure of 337 days), progression in MELD and Child-Pugh scores, and treatment-emergent adverse events were similar among treatment groups.
**Gracia-Tsao et al. (25)**	23	Open-label single-arm clinical trial	Emricasan 25 mg twice daily for 28 days	None	Patients with compensated cirrhosis and PH (HVPG >5 mmHg).	Patients <18, decompensated cirrhosis defined by clinically overt ascites (requiring diuretics), overt encephalopathy (grade II or higher and requiring therapy), or history of variceal hemorrhage. Other exclusions included Child-Pugh class C, other non-liver organ failure, total bilirubin >12 mg/dL, INR >2.5, platelets <20x10^9^/L, overt hepatic encephalopathy of grade III or higher, serum creatinine >2 mg/dL, use of non-selective beta blockers, carvedilol or nitrates, known HIV infection, pancreatitis, portal vein thrombosis, subjects planning to receive anti-HCV therapy during the study, subjects with HBV on stable therapy for less than 3 months, or hepatocellular carcinoma. An attempt was made to primarily enroll subjects with a history of NASH and/or hepatitis C virus (HCV) related cirrhosis and portal hypertension.	28 days	1 case	Serum cCK18 and caspase-3/7 decreased significantly	There was no significant change in HVPG after emricasan (mean [SD] -1.1[4.57] mmHg). No significant changes in blood pressure or heart rate were noted after Emricasan treatment.
**Harrison et al. (24)**	318	A randomized, placebo-controlled trial	Emricasan 5 mg (107 cases) or 50 mg (106 cases) twice daily for 72 weeks	Placebo (105 cases) twice daily for 72 weeks	Subjects had definite NASH and NASH CRN fibrosis stage F1-F3.	Not specified	76 weeks	33 patients: Intervention (21 cases), placebo (12 cases)	Emricasan did not improve fibrosis without worsening of NASH (Emricasan 5 mg =11.2%; Emricasan 50 mg =12.3%; placebo =19.0%)	Emricasan did not result in NASH resolution without worsening of fibrosis (Emricasan 5 mg =3.7%; Emricasan 50 mg =6.6%; placebo =10.5%).
**Mehta et al. (40)**	23	A randomized, placebo-controlled, phase II trial	High-dose Emricasan (25-50 mg, twice daily) group	Placebo/low-dose Emricasan (5 mg, twice daily) group	Patients (≥18 years of age) with stable compensated or decompensated cirrhosis, presenting with an acute deterioration of liver function and associated organ failure. Cirrhosis was diagnosed by clinical, radiological, and/or histological methods, while acute decompensation was defined as ≤6 weeks.	Recent hospital admission (within 4 weeks) for a complication of cirrhosis, greater than two-organ failure, HIV infection, uncontrolled bacterial infection, pre-existing chronic kidney disease, autoimmune liver disease, active malignancy aside from hepatocellular carcinoma, need for mechanical ventilation, inability to obtain consent, and hemodynamic instability (including use of inotropes, aside from terlipressin for hepatorenal syndrome).	28 days	At 28 days, 14 cases: Intervention (7 cases), control (7 cases)	Emricasan pharmacokinetics: 5 mg dose was associated with low plasma levels (<50 ng/ml), and 25 mg and 50 mg doses showed comparable pharmacokinetic profiles	1- At day 7, no significant differences were noted between placebo/low-dose and high-dose Emricasan groups regarding mean differences in MELD and CLIF-C ACLIF scores, and cCK18/flCK18 (M30/M65). 2- The mean percent reduction in cCK18/M30 in high-dose Emricasan group was significantly higher than the placebo/low-dose group at day 2 (-41.9% *vs* 0, P=0.017) and day 4 (-48.8% *vs* -7.4%, P=0.017).
**Pockros et al. (21)**	105	A non-randomized placebo-controlled trial	Emricasan at various doses (5, 25, 50, 100, 200, and 400 mg) once, twice, or 3 doses/day [14 dosing groups]	Placebo	Patients with ALT or AST elevations between 1.5 and 10 times the upper limit of the normal range and fibrosis stages F0 through F3 on a liver biopsy performed within 36 months of enrollment. NASH was diagnosed with liver biopsies demonstrating at least 1 steatosis and ballooning hepatocytes with inflammation and fibrosis.	Patients with cirrhosis diagnosed upon biopsy or decompensated liver disease	35 days	NR	In patients with HCV, except for 5 mg emricasan, all other doses significantly lowered ALT and AST (*p*=0.0041 to *p*<0.0001 for various dosing groups) compared to placebo	Reduction in aminotransferase activity was seen in patients with NASH but effects were not apparent in the small number of other liver diseases.

NR: Not reported.
